# Comprehensive *in vitro* and *in silico* assessments of metabolic capabilities of 24 genomic variants of *CYP2C19* using two different substrates

**DOI:** 10.3389/fphar.2023.1055991

**Published:** 2023-01-12

**Authors:** Myung-Eui Seo, Byung-Joo Min, Nayoon Heo, Kye Hwa Lee, Ju Han Kim

**Affiliations:** ^1^ Seoul National University Biomedical Informatics (SNUBI), Department of Biomedical Sciences, Seoul National University College of Medicine, Seoul, South Korea; ^2^ National Forensic Service Seoul Institute, Seoul, South Korea; ^3^ Department of Mathematics, University of California, Los Angeles, Los Angeles, CA, United States; ^4^ Department of Information Medicine, Asan Medical Center and University of Ulsan College of Medicine, Seoul, South Korea; ^5^ Seoul National University Biomedical Informatics (SNUBI), Division of Biomedical Informatics, Seoul National University College of Medicine, Seoul, South Korea

**Keywords:** cytochrome P450 2C19 (CYP2C19), mephenytoin, omeprazole, CPIC guidelines, pharmacogenetics, pharmacogenomics, PharmVar, PharmGkB

## Abstract

**Introduction:** Most hepatically cleared drugs are metabolized by cytochromes P450 (CYPs), and Clinical Pharmacogenetics Implementation Consortium (CPIC) guidelines provide curated clinical references for CYPs to apply individual genome data for optimized drug therapy. However, incorporating novel pharmacogenetic variants into guidelines takes considerable time.

**Methods:** We comprehensively assessed the drug metabolizing capabilities of *CYP2C19* variants discovered through population sequencing of two substrates, *S*-mephenytoin and omeprazole.

**Results:** Based on established functional assays, 75% (18/24) of the variants not yet described in Pharmacogene Variation (PharmVar) had significantly altered drug metabolizing capabilities. Of them, seven variants with inappreciable protein expression were evaluated as protein damaging by all three *in silico* prediction algorithms, Sorting intolerant from tolerant (SIFT), Polymorphism Phenotyping v2 (PolyPhen-2), and Combined annotation dependent depletion (CADD). The five variants with decreased metabolic capability (<50%) of wild type for either substrates were evaluated as protein damaging by all three *in silico* prediction algorithms, except CADD exact score of NM_000769.4:c.593T>C that was 19.68 (<20.0). In the crystal structure of the five polymorphic proteins, each altered residue of all those proteins was observed to affect the key structures of drug binding specificity. We also identified polymorphic proteins indicating different tendencies of metabolic capability between the two substrates (5/24).

**Discussion:** Therefore, we propose a methodology that combines *in silico* prediction algorithms and functional assays on polymorphic CYPs with multiple substrates to evaluate the changes in the metabolism of all possible genomic variants in CYP genes. The approach would reinforce existing guidelines and provide information for prescribing appropriate medicines for individual patients.

## 1 Introduction

Genetic polymorphisms are the primary cause of inter-individual variations in drug metabolism (Evans and McLeod, 2003; Ingelman-Sundberg, 2004). The major enzyme family capable of catalyzing drug metabolism is cytochromes P450 (CYPs) (Zanger and Schwab, 2013), and numerous studies have investigated the relationship between genomic variants of CYPs and drug metabolism. With improvements in genotyping and sequencing technologies, these studies have become more common, and pharmacogenomic guidelines for clinical use have become more widely available. Currently, Clinical Pharmacogenetics Implementation Consortium (CPIC) guidelines provide finely curated genetic information based on standard operating procedures (SOP) that detail the procedures and methods of performance for consistently implementing the process according to standardized methods (Robarge et al., 2007). Pharmacogenomics Knowledgebase (PharmGKB) (Whirl-Carrillo et al., 2012) and Pharmacogene Variation (PharmVar) (Gaedigk et al., 2018) are classified as approved authoritative resources for CYPs and other pharmacogenomic variants in CPIC SOP. Since 2018, PharmVar Consortium has operated PharmVar database to collect and genomic variants in pharmacogenes, and genomic information from numerous research results has been reported (Gaedigk et al., 2018). Nevertheless, updating information on the correlations between newly discovered genetic variants and drug metabolism takes considerable time. This is in stark contrast to the numerous genomic variants detected in pharmacogenes occurring over a short time through various studies. This limited knowledge prevents proper assessment of the clinical relevance of various genomic variants. Therefore, a new methodology is needed to analyze the correlation between the variants and drug metabolism and derive the importance of each variant by selecting clinically valuable variants among the reported genomic variants.

Most hepatically cleared drugs (78%) are metabolized by CYP enzymes (Zanger et al., 2008), and cytochrome P450 family 2 subfamily C member 19 (*CYP2C19* [MIM: 124020] (Online Mendelian Inheritance in Man, 2019)) is well known for its genomic variability contributing to its enzyme activity (Evans and Relling, 1999; Zanger et al., 2008; Tornio and Backman, 2018). *CYP2C19* is a member of the cytochrome P450 superfamily of enzymes located on chromosome *10q23.33*. It is involved in the metabolism of various drugs, such as proton pump inhibitors (PPIs) (Li et al., 2004), mephenytoin (de Morais et al., 1994), antidepressants (Brosen, 2004), benzodiazepines (Yasumori et al., 1993), and antiplatelet prodrug clopidogrel (Hulot et al., 2006). Highly polymorphic DNA sequences of *CYP2C19* may account for the correlation between variability in drug metabolism involving *CYP2C19*. Various studies have been conducted to apply *CYP2C19* genotypes to individual clinical management and CPIC guidelines provide well-curated genotype-phenotype information for previously reported genomic variants (Lee, 2012; Scott et al., 2012). The Association for Molecular Pathology (AMP) Pharmacogenomics (PGx) Working Group classifies well-known variants as Tier 1 variants, proposing them as priority in clinical tests (Pratt et al., 2018). However, genomic variants that do not have sufficient information about their correlation with protein functions continue to be detected due to the high polymorphism of *CYP2C19*. Therefore, we consider applying our methodology to these variants.

In the present study, we selected genomic variants of *CYP2C19* from 2,504 representative population subjects in the 1000 Genomes Project, not yet considered in PharmVar (Gaedigk et al., 2018). We also assessed the drug metabolism of four CYP2C19 variants, which designated one of the star alleles that assigned to the 2,504 cohorts. We comprehensively characterized the genomic variants of *CYP2C19* metabolizing enzyme activity for recombinant CYP2C19 using two substrates, *S*-mephenytoin and omeprazole.

## 2 Materials and methods

### 2.1 CYP2C19 phenotype mapping

The variant call format (VCF) files of 2,504 cohorts in the 1000 Genomes Project phase III database (Genomes Project et al., 2015) were downloaded, and haplotypes of each sample were inferred using PHASE 2.1.1 (Stephens et al., 2001; Stephens and Scheet, 2005). We extracted the star alleles of each haplotype that matched the allele definition table of *CYP2C19* sourced from PharmVar released in November 2018 (Gaedigk et al., 2018). The diplotype of each subject was translated into the CYP2C19 phenotype on the diplotype-phenotype table of *CYP2C19* provided by PharmGKB (Whirl-Carrillo et al., 2012).

Subjects were assigned to the ultra rapid metabolizer (UM), rapid metabolizer (RM), normal metabolizer (NM), intermediate metabolizer (IM), poor metabolizer (PM), possible IM, possible PM, or indeterminate (Table 1).

**TABLE 1:** CYP2C19 phenotype and diplotype of 2,504 substrates in the 1000 Genomes Project.

CYP2C19 phenotype	Number of substrates (%)	Diplotype of the substrates determined as corresponding CYP2C19 phenotype
Ultra rapid metabolizer	73 (2.92%)	*17|*17
Rapid metabolizer	412 (16.45%)	*1|*17, *13|*17
Normal metabolizer	810 (32.35%)	*1|*1, *1|*13
Intermediate metabolizer	814 (32.51%)	*1|*3, *1|*4, *2|*1, *2|*13, *2|*17, *3|*17, *35|*1, *35|*13, *35|*17
Poor metabolizer	178 (7.11%)	*2|*2, *2|*3, *2|*35, *3|*3, *35|*35
Possible intermediate metabolizer	11 (.44%)	*1|*9, *9|*13, *9|*17
Possible poor metabolizer	1 (.04%)	*2|*9
Indeterminate	99 (3.95%)	*1|*27, *2|*27, *9|*27, *13|*27, *27|*17, *27|*27, *35|*27
Unknown	106 (4.23%)	*1|unk, *2|unk, *9|unk, *13|unk, *17|unk, *27|unk, *35|unk, unk|*1, unk|*13, unk|*17, unk|*27, unk|*3, unk|unk
Total	2,504 (100%)	.

unk, unknown.

### 2.2 Variant selection

**FIGURE 1:**
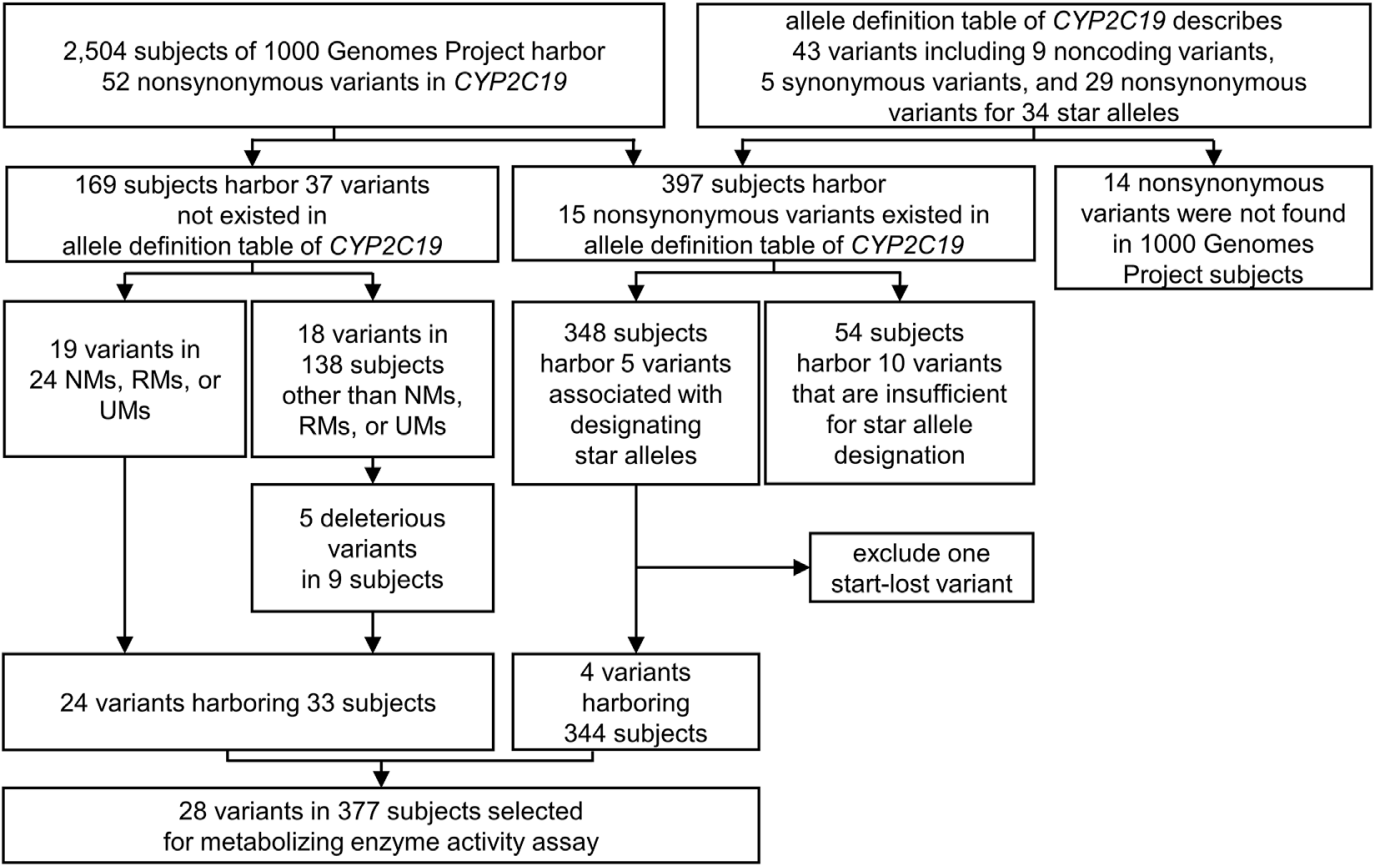
The variant selecting process for metabolizing enzyme activity assay. All the variants in the *CYP2C19* region (*chr10:96522438-96615304*, GRCh37) of 2,504 subjects in the 1000 Genomes Project were considered to select metabolizing capability assay candidates. 24 non-synonymous variants not exist in allele definition table and four non-synonymous variants included in the result of CYP2C19 phenotype classification were selected. UM, Ultra rapid metabolizer; RM, Rapid metabolizer; NM, Normal metabolizer.

We selected the assay candidates from all variants in the *CYP2C19* region (*chr10:96522438-96615304*, GRCh37) of 2,504 cohorts in the 1000 Genomes Project phase III database (Genomes Project et al., 2015) to measure the metabolic capability of the variants that occur spontaneously (Figure 1). These variants were divided into two groups based on their presence in the allele definition table provided by PharmGKB (Whirl-Carrillo et al., 2012), sourced from PharmVar released in November 2018 (Gaedigk et al., 2018). Among the variants not presented in the table, we chose non-synonymous variants for mutagenesis. A total of 37 missense variants were selected, and 169 subjects were confirmed to have these variants. Of these variants, we selected all those identified in NMs, RMs, or UMs (24 subjects), and all 19 variants were selected for the assay (Figure 1; Table 2). The remaining 145 subjects had 24 variants, and 18 variants remained after excluding those in NMs, RMs, or UMs. Of these 18 variants, we chose deleterious variants that satisfied the following criteria:

SIFT=0∩PolyPhen−2=1∩CADD>20

**TABLE 2:** 28 genomic variants of *CYP2C19* selected for metabolizing enzyme activity assay.

Variant (GenBank: NM_000769.4)	rs ID	ACMG/AMP	SIFT	PolyPhen-2	CADD	1000g allele frequency	Other non-synonymous variants found on the same allele as the novel variant in 33 subjects
24 genomic variants of *CYP2C19* harboring 33 subjects							
c.124A>G [p.Ile42Val]	rs559087813	VUS	.33	.00	6.33	.0002	.
c.164C>G [p.Thr55Ser]	rs572853437	VUS	.85	.00	.00	.0008	.
c.218G>A [p.Arg73His]	rs201306972	VUS	.01	.02	8.90	.0006	.
c.221T>C [p.Met74Thr]	rs28399505	VUS	.70	.00	.03	.0008	.
c.326G>C [p.Gly109Ala]	rs200347843	VUS	.01	.05	12.76	.0004	.
c.373C>T [p.Arg125Cys]	rs200150287	VUS	.01	.99	23.70	.0002	.
c.389C>T [p.Thr130Met]	rs150152656	VUS	.00	1.00	22.10	.0002	.
c.394C>T [p.Arg132Trp]	rs149590953	VUS	.00	.99	21.90	.0006	.
c.478A>G [p.Lys160Glu]	rs375760063	VUS	.21	.02	15.59	.0002	.
c.556C>T [p.Arg186Cys]	rs183701923	VUS	.02	1.00	24.80	.0004	.
c.593T>C [p.Met198Thr]	rs186489608	VUS	.01	.26	19.68	.0002	.
c.738G>T [p.Glu246Asp]	rs574458036	VUS	.09	.00	4.46	.0002	.
c.778C>A [p.Pro260Thr]	rs556994963	VUS	.00	1.00	23.90	.0002	.
c.784G>A [p.Asp262Asn]	rs577255883	VUS	.06	.68	24.90	.0006	.
c.831C>A [p.Asn277Lys]	rs559628884	VUS	.06	.29	4.11	.0002	.
c.837G>T [p.Gln279His]	rs547822797	VUS	.44	.00	8.78	.0002	.
c.985C>T [p.Arg329Cys]	rs59734894	VUS	.06	.49	13.32	.0002	.
c.1003C>T [p.Arg335Trp]	rs368758960	VUS	.00	1.00	23.10	.0002	.
c.1034T>A [p.Met345Lys]	rs201132803	VUS	.00	1.00	25.20	.0002	.
c.1150G>C [p.Gly384Arg]	rs188851578	VUS	.01	1.00	26.50	.0002	.
c.1160T>C [p.Ile387Thr]	rs562912432	VUS	.00	1.00	22.20	.0002	.
c.1295A>T [p.Lys432Ile]	rs146991374	VUS	.00	1.00	23.00	.0010	.
c.1330G>C [p.Glu444Gln]	rs540369401	VUS	.00	1.00	26.20	.0002	.
c.1465C>T [p.Pro489Ser]	rs542090374	VUS	.00	.98	19.28	.0002	.
Four genomic variants associated with star alleles							
c.431G>A [p.Arg144His] (CYP2C19*9)	rs17884712	VUS	.01	1.00	23.70	.0028	.
c.636G>A [p.Trp212Ter] (*CYP2C19* *3)	rs4986893	VUS	N.A.	N.A.	34.00	.0142	.
c.991G>A [p.Val331Ile] (CYP2C19*38)	rs3758581	VUS	1.00	.01	.00	.0485	.
c.1228C>T [p.Arg410Cys] (CYP2C19*13)	rs17879685	LB	.01	.00	16.96	.0056	.

LB, likely benign; VUS, variant of uncertain significance; N.A., not available.

Five [GenBank (Benson et al., 2018): NM_000769.4:c.389C>T, c.778C>A, c.1003C>T, c.1160T>C, and c.1295A>T] variants in nine subjects were selected for the assay (Figure 1; Table 2).

Of the total non-synonymous variants of the *CYP2C19* region in the 1000 Genomes Project phase III database (Genomes Project et al., 2015), five variants were identified as present in the allele definition table sourced from PharmVar released in November 2018 (Gaedigk et al., 2018), while at the same time designating one of the star alleles assigned to the 2,504 cohorts (Figure 1; Table 1). The five variants NM_000769.4:c.1A>G, c.431G>A, c.636G>A, c.991G>A, and c.1228C>T, are the sole non-synonymous variants associated with CYP2C19*4, *9, *3, *38, and *13, respectively. Therefore, enzyme activity was measured for all four variants except NM_000769.4:c.1A>G, the start-lost variant (Figure 1; Table 2). One of the four selected variants was NM_000769.4:c.636G>A, which determines that CYP2C19*3, has no function due to truncation. NM_000769.4:c.431G>A is a variant that determines CYP2C19*9, and NM_000769.4:c.1228C>T is CYP2C19*13, known as decreased function and normal function, respectively. The last variant, NM_000769.4:c.991G>A, is associated with most star alleles. The variant defines the allele originally cataloged as CYP2C19*1.001, and the allele is classified as normal function. It has now received CYP2C19*38 (Botton et al., 2021). The number of subjects with these four variants was 344. Finally, 28 variants in 377 subjects were selected for metabolizing enzyme activity assay (Figure 1; Table 2).

### 2.3 Expression and purification of polymorphic P450 2C19 for functional assay

The open reading frame (ORF) of wildtype (WT) *CYP2C19* (GenBank: NM_000769.4) was synthesized and cloned into the mammalian expression vector pcDNA™3.4 with HindⅢ site. Site-direct mutagenesis was performed based on the WT expression plasmid and verified by Sanger sequencing (Supplementary Figure S1). WT and mutant carrying pDNA were transfected into Expi293F cells with 1 L culture volume scale by using Expi293™ Expression System kit and harvested at 72 h post transfection. A small part of the cells (5 mL) was separated and the cell lysate identified sufficient amounts of cell expression through SDS-polyacrylamide gel electrophoresis (SDS-PAGE). All materials were from ThermoFisher Scientific (Waltham, MA, United States), and all processes followed the manufacturer’s instructions.

The remaining cells were washed twice with .1 M of potassium phosphate buffer (pH 7.4), and resuspended in a lysis buffer containing 100 mM phosphate (pH 7.4), 1 mM ethylenediaminetetraacetic acid, 1 mM dithiothreitol, .1 mM phenylmethane sulfonyl fluoride, and 20% glycerol. Microsomes with recombinant CYP2C19 were extracted by centrifugation at 12,000 × g for 10 min at 4°C. The final products were pelleted by centrifugation at 100,000 × g for 1 h at 4°C. Microsomal pellets were resuspended in storage buffer [100 mM phosphate (pH 7.4), 1 mM ethylenediaminetetraacetic acid, and 20% glycerol], and stored at −80°C until use.

The microsomal fraction from human embryonic kidney cells was separated on a 10% SDS-PAGE to detect the recombinant CYP2C19 expression. The gels were then transferred to a polyvinylidene difluoride (PVDF) membrane and at 85 V for 90 min by using a semi-dry apparatus (Bio-Rad, Hercules, CA, United States). The membrane was immunoblotted with anti-CYP2C19 antibody (ab137015; Abcam Inc., Cambridge, MA, United States) and incubated with horseradish peroxidase-conjugated (HRP) anti-rabbit IgG (ab270144; Abcam Inc., Cambridge, MA, United States). Protein bands were visualized with ECL Western Blotting Detection System (Santa Cruz Biotechnology) and the gel images were investigated by Chemiluminescence imaging system (WSE-6200 LuminoGraph II, ATTO, JAPAN). Commercially available baculosomes coexpressing CYP2C19 and oxidoreductase (SBC02C190; SPMED Co., Ltd., Busan, Republic of Korea) were used as positive control and the empty vector as a negative control, since Expi293F™ cells do not express CYP2C19 (Uhlen et al., 2017).

P450 2C19 was monitored by CO-difference spectrum to quantitate specific P450 that functions as the cytochrome P450 family using a UV visible spectrophotometer (UV-1650PC, SHIMADZU, Japan) (Omura and Sato, 1964). Microsomal preparations containing 2 mg of total protein were placed in both the sample and reference cuvettes, and the baseline was recorded between 400 and 500 nm. The sample cuvette was then treated with CO for 40 s and the spectral difference was measured between 400 and 500 nm after the reduction of both cuvettes with 1 mg of solid sodium dithionite. The P450 contents was calculated from the absorbance at 450 and 490 nm using the following formula:

$$\left(∆A450-∆A490\right)/0.091=nmol\;ofP450/mL$$

Specific P450 contents were calculated using the following formula:

$$P450\;contents\left(nmol/mL\right)/protein\;\;concentration\left(mg\;\;∕mL\right)=specific\;P450\;contents\left(nmol/mgprotein\right)$$

### 2.4 Metabolizing enzyme activity assay for recombinant P450 2C19

*S*-mephenytoin and omeprazole, *in vitro* markers and clinical substrates for CYP2C19-mediated metabolism designated by the United States Food and Drug Administration (U.S. FDA) (U S Food and Drug Administration, 2022), were used for measuring metabolizing enzyme activity. The metabolite of each substrate was determined using liquid chromatography–tandem mass spectrometry (LC-MS/MS) with its authentic standard. To measure OH-mephenytoin production, 40 pmol of recombinant P450 2C19 was mixed with 100 mM phosphate buffer and 100 μM *S*-mephenytoin and pre-incubated at 37°C for 5 min. The reaction was performed with nicotinamide adenine dinucleotide phosphate (NADPH) regenerating system (1.3 mM of b-NADP+, 3.3 mM of glucose 6-phosphate, .1 U/mL of glucose 6-phosphate dehydrogenase, 3.3 mM of magnesium chloride) for 30 min at 37°C. The reaction was stopped on ice by adding acetonitrile containing 2.5 μM of chlorpropamide, and centrifuged at 16,000 × g for 5 min at 4°C. Omeprazole was used in the same way, except that 100 pmol recombinant P450 2C19 enzymes were incubated with 20 μM omeprazole for 45 min. The supernatant was injected into Agilent 6410 LC-MS/MS system (Agilent, Wilmington, DE, United States) and separated on Kinetex C18 (50 × 2.1 mm i.d., 2.6 μm; Phenomenex^®^, Torrance, CA, United States) in mobile phase comprised (A) distilled water containing .1% formic acid and (B) 100% acetonitrile containing .1% formic acid at a flow rate of 200 μL/min for 5 min. The mass spectra of the two substrates and their respective metabolites were recorded by electrospray ionization in the positive ion mode. The turbo-ion spray interface was operated in the positive mode at 5,000 V and 400°C. The optimum collision energies for the ionization of OH-mephenytoin and 5′-hydroxy omeprazole were 17 and 10 eV, respectively. Multiple reaction monitoring modes using specific precursor-to-product ion transitions were applied for quantification. *S*-mephenytoin was detected at ion transitions of 230 m/z → 150 m/z, whereas omeprazole was detected at the transitions of 362 m/z → 214 m/z. The limit of quantification (LLOQ) of the assay was .02 μM for *S*-mephenytoin and .01 μM for omeprazole. LC-MS/MS were performed using the and procedures provided by SPMED Co., Ltd., Busan, Republic of Korea. All experiments were done in triplicate and the mean value ± standard deviation (SD) of the measured results was used. The statistical levels of the metabolizing capability differences between the two substrates in each polymorphic CYP2C19 were calculated by independent two sample *t*-test using R, and *p* < .05 was considered to indicate statistical significance.

### 2.5 Homology stereoscopic model of CYP2C19 with variants

The crystal structure of human CYP2C19 was generated using the atomic coordinate set structure of human microsomal cytochrome P450 2C19 [PBD: 4GQS (Reynald et al., 2012)] in Protein Data Bank (PDB) with PyMOL (Schrodinger, 2021). Six substrate recognition sites (SRSs), the important regions the in binding of substrates in CYPs (Gotoh, 1992), were presented on 4GQS (Reynald et al., 2012) following previous studies (Figure 4) (Gotoh, 1992; Sirim et al., 2010; Nair et al., 2016; Derayea et al., 2019; Zhang et al., 2020). We rendered the stereoscopic structure of CYP2C19 carrying each variant with less than 50% of WT metabolism on either substrate, and observed changes in the surrounding structure, adjacent SRSs, or the active site (Otyepka et al., 2007).

## 3 Results

### 3.1 Selection of genomic variants from the subjects of the 1000 Genomes Project

The 2,504 cohorts were classified as 810 NMs (32.35%), 73 UMs (2.92%), 412 RMs (16.45%), 814 IMs (32.15%), 178 PMs (7.11%), 11 possible IMs (.44%), one possible PMs (.04%), 106 unknown metabolizers (4.23%), and 99 indeterminable subjects (3.95%) (Table 1).

We selected 24 genomic variants not present in the allele definition table, but identified in 33 subjects of the 1000 Genomes Project for metabolizing enzyme activity assay. No other non-synonymous variant was found on the same allele in which each variant was located in 33 subjects (Figure 1; Table 2). NM_000769.4:c.431G>A, c.636G>A, c.991G>A, and c.1228C>T associated with CYP2C19*9, *3, *38, and *13, respectively, were also selected, with variants was identified in 344 subjects (Figure 1; Table 2). Thus, 28 variants were selected from 2,504 subjects from the 1000 Genomes Project.

### 3.2 Detection of recombinant CYP2C19 expression using a human cell system

**FIGURE 2:**
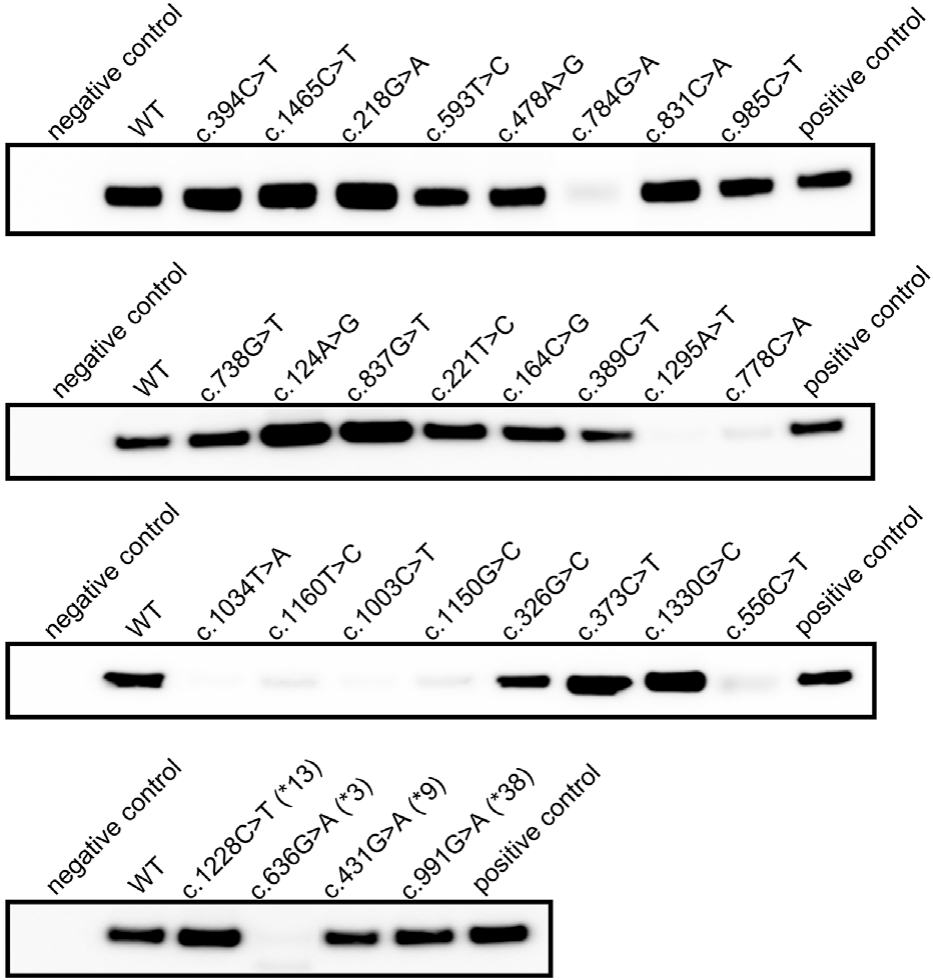
Immunoblot result of recombinant CYP2C19 extracted from human microsomal membrane. Immunoblot analysis of recombinant CYP2C19 extracted from microsomal extracts. SPMED™ Human Recombinant Enzymes CYP2C19 (SBC02C190, SPMED Co., Ltd., Busan, South Korea) was used as positive control and empty vector as negative control.

Proteins were obtained by microsome extraction, and immunoblot assay was performed. The results confirmed that CYP2C19 containing c.636G>A (CYP2C19*3) matched the negative control, and WT matched the positive control (Figure 2). Despite the absence of non-sense mutation, the recombinant proteins for each of the eight variants revealed inappreciable protein expression (c.556C>T, c.778C>A, c.784G>A, c.1003C>T, c.1034T>A, c.1150G>C, c.1160T>C, and c.1295A>T) (Figure 2). The remaining recombinant proteins were fully expressed in human microsomes.

The specific P450 content calculated from the CO-difference spectrum results also demonstrated that truncated CYP2C19 and WT represent negative and positive controls, respectively (Table 3). Spectral peaks at approximately 450 nm were not readily detected in the microsomal fraction from the cells transfected with each of the eight individual variants, indicating low protein expression in the immunoblot assay (Figure 2; Table 3). Immunoblot results identified sufficient protein expression for the microsome extracts obtained from c.218G>A expressing cells, but the absorption peak at approximately 450 nm was not readily detected (Figure 2; Table 3). A total of 19 recombinant proteins including WT were confirmed to have sufficient P450 2C19 for LC-MS/MS analysis (Table 3).

**TABLE 3:** Results of specific P450 measurement and metabolizing capability assay for mephenytoin and omeprazole.

Variant (GenBank: NM_000769.4)	Specific P450 content (pmol/mg protein)	OH-mephenytoin formation^a^ (pmol/min/pmol P450) (%WT)	5-Hydroxy omeprazole formation^a^ (pmol/min/pmol P450) (%WT)	ACMG/AMP	SIFT	PolyPhen-2	CADD
c.124A>G [p.Ile42Val]	96.7	.24 ± .03 (103.9%)	4.41 ± .31 (104.2%)	VUS	Tolerated	Benign	6.33
c.164C>G [p.Thr55Ser]	71.6	.36 ± .06 (155.6%)	5.11 ± .37 (120.7%)	VUS	Tolerated	Benign	.00
c.218G>A [p.Arg73His]	N.D.	N.D. (N.A.)	N.D. (N.A.)	VUS	Damaging	Benign	8.90
c.221T>C [p.Met74Thr]	429.3	.28 ± .03 (119.1%)	2.88 ± .07 (67.9%)	VUS	Tolerated	Benign	.03
c.326G>C [p.Gly109Ala]	25.0	.21 ± .00 (90.1%)	6.44 ± .43 (152.2%)	VUS	Damaging	Benign	12.76
c.373C>T [p.Arg125Cys]	128.6	N.D. (N.A.)	.96 ± .10 (22.6%)	VUS	Damaging	Probably damaging	23.70
c.389C>T [p.Thr130Met]	46.0	.04 ± .01 (15.5%)	3.67 ± .08 (86.8%)	VUS	Damaging	Probably damaging	22.10
c.394C>T [p.Arg132Trp]	190.1	N.D. (N.A.)	.48 ± .04 (11.3%)	VUS	Damaging	Probably damaging	21.90
c.431G>A [p.Arg144His] (CYP2C19*9)	163.2	.07 ± .00 (31.8%)	2.67 ± .22 (63.1%)	VUS	Damaging	Probably damaging	23.70
c.478A>G [p.Lys160Glu]	206.8	.22 ± .01 (94%)	4.41 ± .13 (104.1%)	VUS	Tolerated	Benign	15.59
c.556C>T [p.Arg186Cys]	N.D.	N.D. (N.A.)	N.D. (N.A.)	VUS	Damaging	Probably damaging	24.80
c.593T>C [p.Met198Thr]	51.5	.12 ± .01 (49.6%)	3.44 ± .25 (81.2%)	VUS	Damaging	Possibly damaging	19.68
c.636G>A [p.Trp212Ter] (CYP2C19*3)	N.D.	N.D. (N.A.)	N.D. (N.A.)	VUS	N.A.	N.A.	34.00
c.738G>T [p.Glu246Asp]	140.5	.23 ± .03 (97.8%)	4.11 ± .05 (97%)	VUS	Tolerated	Benign	4.46
c.778C>A [p.Pro260Thr]	N.D.	N.D. (N.A.)	N.D. (N.A.)	VUS	Damaging	Probably damaging	23.90
c.784G>A [p.Asp262Asn]	N.D.	N.D. (N.A.)	N.D. (N.A.)	VUS	Tolerated	Possibly damaging	24.90
c.831C>A [p.Asn277Lys]	123.2	.30 ± .04 (126.8%)	4.81 ± .39 (113.7%)	VUS	Tolerated	Possibly damaging	4.11
c.837G>T [p.Gln279His]	211.3	.29 ± .05 (122.8%)	4.48 ± .05 (105.8%)	VUS	Tolerated	Benign	8.78
c.985C>T [p.Arg329Cys]	96.5	.39 ± .05 (165.2%)	4.88 ± .22 (115.4%)	VUS	Tolerated	Possibly damaging	13.32
c.991G>A [p.Val331Ile] (CYP2C19*38)	167.0	.09 ± .01 (37.8%)	2.96 ± .09 (70%)	VUS	Tolerated	Benign	.00
c.1003C>T [p.Arg335Trp]	N.D.	N.D. (N.A.)	N.D. (N.A.)	VUS	Damaging	Probably damaging	23.10
c.1034T>A [p.Met345Lys]	N.D.	N.D. (N.A.)	N.D. (N.A.)	VUS	Damaging	Probably damaging	25.20
c.1150G>C [p.Gly384Arg]	N.D.	N.D. (N.A.)	N.D. (N.A.)	VUS	Damaging	Probably damaging	26.50
c.1160T>C [p.Ile387Thr]	N.D.	N.D. (N.A.)	N.D. (N.A.)	VUS	Damaging	Probably damaging	22.20
c.1228C>T [p.Arg410Cys] (CYP2C19*13)	195.6	.23 ± .03 (100.1%)	4.13 ± .25 (97.5%)	LB	Damaging	Benign	16.96
c.1295A>T [p.Lys432Ile]	N.D.	N.D. (N.A.)	N.D. (N.A.)	VUS	Damaging	Probably damaging	23.00
c.1330G>C [p.Glu444Gln]	299.1	.04 ± .002 (15.9%)	1.77 ± .05 (41.9%)	VUS	Damaging	Probably damaging	26.20
c.1465C>T [p.Pro489Ser]	26.3	.41 ± .07 (174.7%)	11.38 ± .53 (269%)	VUS	Damaging	Probably damaging	19.28
WT	208.2	.23 ± .004 (100%)	4.23 ± .05 (100%)	.	.	.	.

^a^ The mean value ± SD, *p-values<.05, N.A., not available; N.D., not detected; LB, likely benign; VUS, variant of uncertain significance.

### 3.3 Estimation of metabolic capabilities of P450 2C19 with variants for *S*-mephenytoin

The metabolizing enzyme activities of recombinant P450 2C19 in the biotransformation of S-mephenytoin to OH-mephenytoin were measured (Table 3). P450 2C19 containing c.431G>A (CYP2C19*9) indicated 31.8% of WT metabolism, c.991G>A (CYP2C19*38) revealed 37.8% of WT metabolism, and c.1228C>T (CYP2C19*13) indicated 100.1% of WT metabolism. The assay results identified that the metabolizing capability of c.431G>A (CYP2C19*9) and c.1228C>T (CYP2C19*13) were consistent with their CPIC clinical function, similar to c.636G>A (CYP2C19*3) which classified as no function.

Of the 14 polymorphic CYP2C19, the production of OH-mephenytoin was not observed from the P450 2C19 carrying c.373C>T or c.394C>T. The metabolic capability of P450 2C19 containing c.389C>T or c.1330G>C was 15.5% and 15.9% of WT metabolism, respectively. The results for these four variants were lower than those of c.431G>A (CYP2C19*9). There were three variants exhibited rapid metabolizing enzyme activity over 150% of WT. Of these, the result of c.1465C>T (174.7%) was nearly twice higher compared to WT. The remaining seven polymorphic P450 2C19 were confirmed to have biotransformation capability close to WT, except for that carrying c.593T>C with 49.6% of WT metabolism.

### 3.4 Estimation of metabolic capabilities of P450 2C19 with variants for omeprazole

The metabolizing enzyme activity of P450 2C19 containing each variant was compared with that of the WT by measuring the production of 5-hydroxy omeprazole using omeprazole as a substrate (Table 3). P450 2C19 with c.431G>A (CYP2C19*9), c.991G>A (CYP2C19*38) or c.1228C>T (CYP2C19*13) were observed to have 63.1%, 70.0% or 97.5% of WT metabolism, respectively. The assay results of P450 2C19 carrying c.991G>A (CYP2C19*38) or c.1228C>T (CYP2C19*13) were consistent with their CPIC clinical function, normal function.

Remarkably low concentrations of 5-hydroxy omeprazole were detectable for c.394C>T (11.3%) and c.373C>T (22.6%). The P450 2C9 carrying c.1330G>C also revealed a low metabolizing capability, which was 41.9% of WT metabolism. Two polymorphic P450 2C19 indicated a faster metabolic rate over 150% of WT. The P450 2C19 containing c.1465C>T was measured to have a three times higher metabolic capability (269.0%) than WT metabolism. The assay results of this variants was over 150% of WT metabolism in both substrates. The other one (c.326G>C) indicated 152.2% of WT metabolism for omeprazole but approximated WT for *S*-mephenytoin. The assay results of ten variants were similar to that of WT metabolism. Among them, two of which had a metabolic capability of less than 50% of WT metabolism (c.389C>T and c.593T>C), and two were over 150% (c.164C>G and c.985C>T) to *S*-mephenytoin.

### 3.5 Evaluation of metabolizing capability difference between the two substrates

**FIGURE 3:**
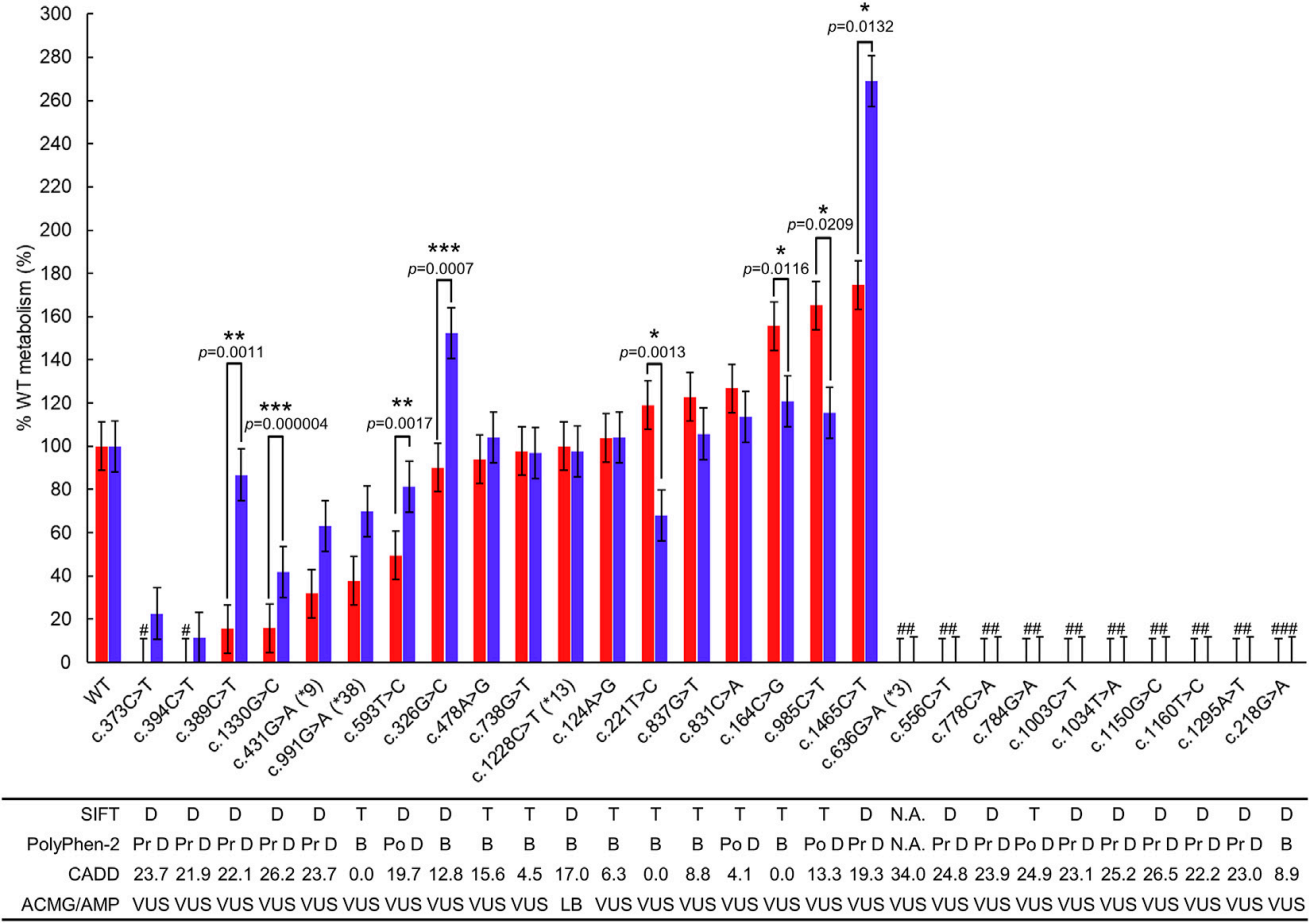
Metabolizing capability compared to WT P450 2C19 metabolism of mephenytoin and omeprazole. The metabolic capability of each polymorphic P450 2C19 was compared with WT using OH-mephenytoin (red bar) or 5-hydroxy omeprazole (blue bar) formation. Each bar represents %WT ± SE of the polymorphic P450 2C19 containing the variant. Variants with SIFT score less than .05 are predicted to be deleterious (D), those greater than or equal to .05 are predicted to be tolerated (T) as described in SIFT algorithm. If the PolyPhen-2 score is between the following intervals (0, .2), (.2, .85), and (.85, 1), the scores are predicted to be benign (B), possibly damaging (Po D) and probably damaging (Pr D), respectively, as described in the algorithm. For the variants indicated significantly different metabolizing capability between the two substrates, *p*-values < .05, *p*-values < .01, and *p*-values < .001 are marked as *, **, and ***, respectively. ^#^The formation of metabolites was not detectable. ^##^The expression of polymorphic CYP2C19 containing the variant was not detectable in the immunoblotting. ^###^The spectral peaks at approximately 450 nm were not readily detected in the microsome extracts. LB, Likely Benign; VUS, Variant of Uncertain Significance.

We analyzed metabolic capability differences between the two substrates for each polymorphic CYP2C19, and identified eight polymorphic CYP2C19 with a difference of more than 25% (Table 3; Figure 3). Of these eight proteins, the largest metabolic capability difference (94.3%, *p* = .0132) was observed in the case of polymorphic P450 2C19 containing c.1465C>T. However, the metabolic capabilities to the two substrates were both more than 150% of WT metabolism (*S*-mephenytoin 174.7%, omeprazole 269.0%). The metabolic capabilities of polymorphic P450 2C19 containing c.1330G>C for the two substrates were both less than 50% of WT metabolism (*S*-mephenytoin 15.9%, omeprazole 41.9%, *p* = .000004), and polymorphic P450 2C19 containing c.221T>C were both 50%–150% metabolic capability of WT (*S*-mephenytoin 119.1%, omeprazole 67.9%, *p* = .0013). There were differences in the amount of change in metabolic capability due to these three polymorphic CYP2C19, but the differences in the tendency were not confirmed.

The remaining five polymorphic CYP2C19 indicated different tendencies of metabolic capability in the two substrates; c.164C>G (*S*-mephenytoin 155.6%, omeprazole 120.7%, *p* = .0116), c.326G>C (*S*-mephenytoin 90.1%, omeprazole 152.2%, *p* = .0007), c.389C>T (*S*-mephenytoin 15.5%, omeprazole 86.8%, *p* = .0011), c.593T>C (*S*-mephenytoin 49.6%, omeprazole 81.2%, *p* = .0017), c.985C>T (*S*-mephenytoin 165.2%, omeprazole 115.4%, *p* = .0209).

### 3.6 Comparison with *in silico* protein damage prediction algorithms

The results of the *in vitro* metabolic enzyme activity targeting P450 2C19 variants were compared with three *in silico* protein damage prediction algorithms: Sorting intolerant from tolerant (SIFT) (Sim et al., 2012), Polymorphism Phenotyping v2 (PolyPhen-2) (Adzhubei et al., 2010), and Combined Annotation Dependent Depletion (CADD) (Rentzsch et al., 2019) (Figure 3; Table 3).

As for the eight variants with low protein expression, all three *in silico* prediction algorithms agreed that the variants caused protein damage except SIFT evaluated c.784G>A as tolerated, despite PolyPhen-2 and CADD evaluating it as possibly damaging, and with an exact score of 24.90.

Of the 18 genomic variants in sufficiently expressed P450 2C19 polymorphic proteins, three were associated with the star allele (c.431G>A (CYP2C19*9), c.991G>A (CYP2C19*38), and c.1228C>T (CYP2C19*13)), and 15 were not. While P450 2C19 containing c.1228C>T (CYP2C19*13) indicated 50%–150% metabolic capability of WT for both substrates, P450 2C19 carrying each of c.431G>A (CYP2C19*9) and c.991G>A (CYP2C19*38) exhibited differential metabolic capabilities for *S*-mephenytoin (less than 50% metabolic capability of WT) and omeprazole (50%–150% metabolic capability of WT). All three *in silico* prediction algorithms predicted c.431G>A (CYP2C19*9) as a protein damage variant and c.991G>A (CYP2C19*38) as a benign variant. Since these *in silico* prediction algorithms provide one predictive value for a single variant, it was impossible to predict the difference in metabolic capacities by substrate type.

Of the 15 variants not present in the allele definition table, all three polymorphic P450 2C19 carrying each of c.373C>T, c.394C>T, and c.1330G>C indicated decreased metabolic capabilities less than 50% of WT for both substrates. These variants were consistently evaluated as protein-damaging variants by all three *in silico* prediction algorithms. Two polymorphic P450 2C19 containing each of c.389C>T and c.593T>C exhibited decreased metabolic capabilities of less than 50% of WT for *S*-mephenytoin but not for omeprazole. All three *in silico* prediction methods evaluated these two variants as protein-damaging variants, except that the CADD exact score for c.593T>C was 19.68. The five variants (c.124A>G, c.221T>C, c.478A>G, c.738G>T, and c.837G>T) in P450 2C19 polymorphic proteins with 50%–150% metabolic capability of WT for *S*-mephenytoin were evaluated as benign by all three *in silico* prediction algorithms, whereas the two (c.326G>C and c.831C>A) were predicted by two as benign and by one as protein damaging.

For omeprazole, polymorphic P450 2C19 proteins containing each of the 10 genomic variants not in the allele definition table exhibited 50%–150% metabolic capability of WT. Of these, as described above, two polymorphic P450 2C19 carrying each of c.389C>T and c.593T>C were evaluated as protein damaging by *in silico* prediction algorithms. Six of the remaining eight genomic variants (c.124A>G, c.164C>G, c.221T>C, c.478A>G, c.738G>T, and c.837G>T) in polymorphic P450 2C19 proteins with 50%–150% metabolizing capabilities of WT were evaluated as tolerated/benign by all three *in silico* prediction algorithms. SIFT and CADD evaluated the remaining two genomic variants (c.831C>A and c.985C>T) as benign; PolyPhen-2 evaluated it as possibly damaging.

P450 2C19 containing each of the four variants (c.164C>G, c.326G>C, c.985C>T, and c.1465C>T) exhibited metabolic capability of 150% or more compared to WT for either substrates. One genomic variant (c.1465C>T) in P450 2C19 with more than 150% metabolic capability of WT for both substrates was consistently evaluated as damaging by all three *in silico* prediction algorithms. Proteins containing the remaining three variants were considered to have more than 150% metabolic capability compared to WT for only one of the two substrates, and *in silico* predictions were also conflicting.

### 3.7 The effects of the variants on stereoscopic structure

**FIGURE 4:**
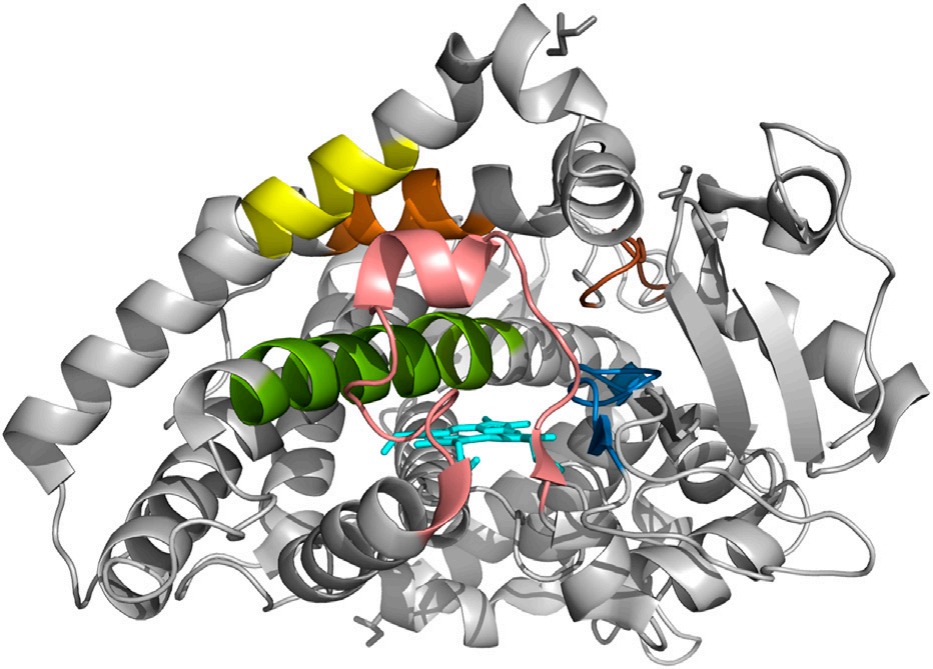
The crystal structure of CYP2C19 with six substrate recognition sites. Molecular structure of CYP2C19 was rendered by using the atomic coordinate set PDB 4GQS. Six SRSs are indicated as follows; SRS 1 in pink, SRS 2 in orange; SRS 3 in yellow, SRS 4 in green, SRS 5 in blue, and SRS 6 in brown. The heme is also illustrated (cyan).

Six SRSs and the active site of CYP2C19 were presented on 4GQS (Reynald et al., 2012) in PDB (Figure 4). The five variants that did not exist in the allele definition table were selected from those resulted in less than 50% of WT metabolism on either substrate. The stereoscopic structure of polymorphic CYP2C19 containing each of these genomic variants were visualized using 4GQS.

**FIGURE 5:**
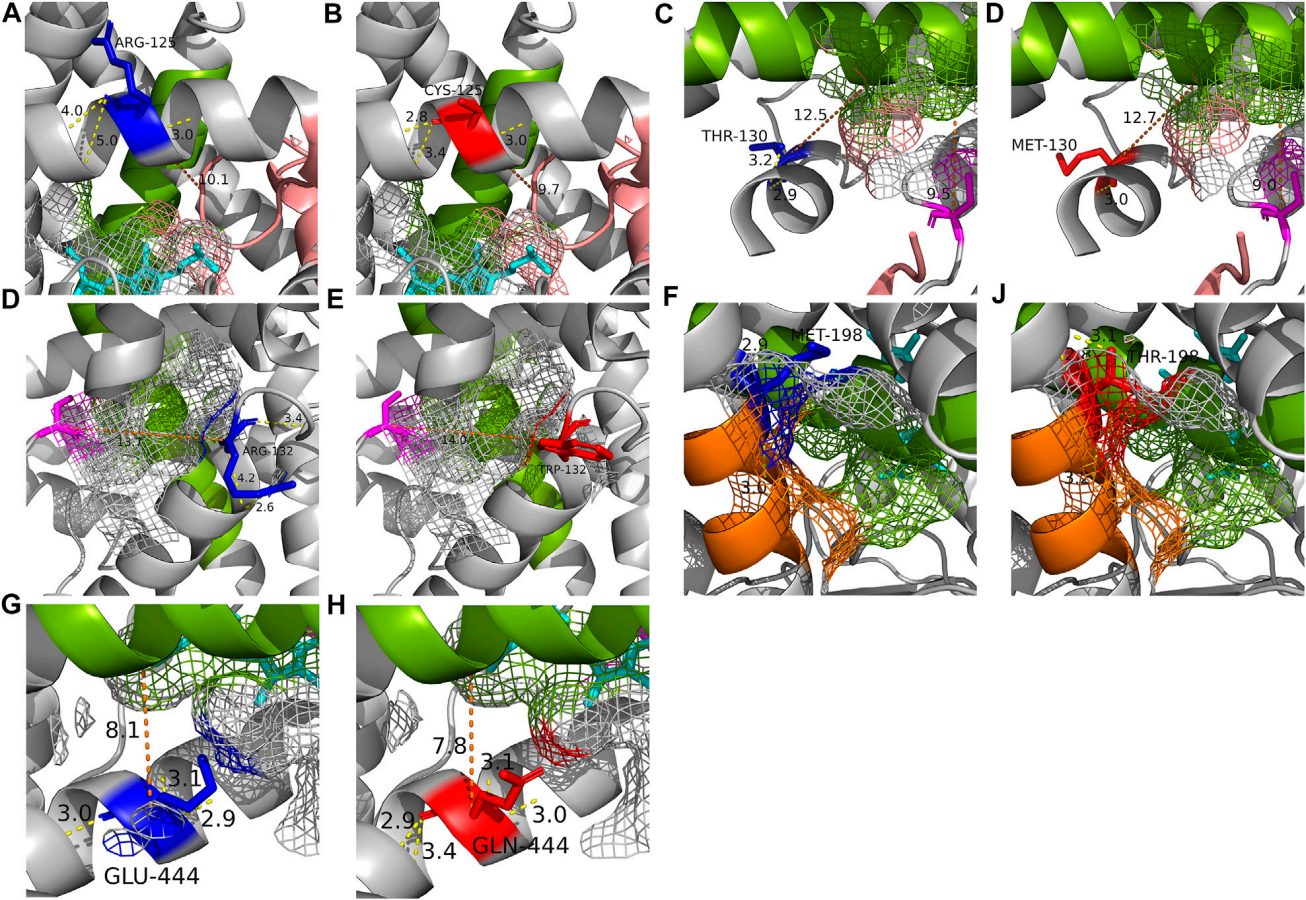
The crystal structure of each of the five altered residues and their surrounding area to compare the structural effects with WT (PDB: 4GQS). **(A, B)** Stereoscopic structures of Arg125 (WT) and Cys125. **(C, D)** Stereoscopic structures of Thr130 (WT) and Met130. **(E, F)** Stereoscopic structures of Arg132 (WT) and Trp132. **(G, H)** Stereoscopic structures of Met198 (WT) and Thr198. **(I, J)** Stereoscopic structures of Glu444 (WT) and Gln444. The blue residue is WT form of the residue and the red is altered form of the residue. The magenta residue is the active site and heme is shown as cyan. The SRSs are shown in the following colors; SRS 1, pink; SRS 2, orange; SRS 4, green. The yellow line is hydrogen bond and the number on it is distance of the bond. The orange line is the distance between two structures.

The alteration of Arg125 to Cys125 revealed a surface change near residue 293 of SRS 4, and alteration in the hydrogen bonds within the helix brought the distance from SRS 1 (Figures 5A, B). The disappearance of a hydrogen bond, distance change from SRS 4, and structural changes between residues 293-301 of SRS 4, which is directly involved in the binding of mephenytoin (Payne et al., 1999), were observed around Met130 compared with Thr130 (Figures 5C, D). It was also observed that the distance between SRS 4 and the active site decreased (Figures 5C, D). The stereoscopic structure around Arg132 was compared with Trp132, showing a change in the distance from the active site (Figures 5E, F). The polar contacts that Arg132 forms with the surrounding residues, disappeared when Arg132 was replaced with Trp132. Met198 was found to exist in SRS 2 (Figure 5G), and the amino acid replacement of Met 198 with Thr198 occurred a new polar contact with helix F, and a gap change in the helix structure of SRS 2 was detected (Figure 5H). Gln444 has been identified as creating a new hydrogen bond and getting closer to SRS 4 compared with Glu444 (Figures 5I, J). The crystal structure of each of the five variants affected the active site or SRS 4, a well-known important structure for discriminating drug specifications of *CYP2C19* (Evans and Relling, 1999; Tornio and Backman, 2018).

## 4 Discussion

In the present study, the metabolizing enzyme activity assay was applied to measure the drug biotransformation capability of *CYP2C19* genomic variants observed in the general population but not considered yet in the allele definition table (Figure 1; Table 2). The non-synonymous variants designating CYP2C19*9, *3, *38, and *13 were also included in the study (Figure 1; Table 2). We compared the evaluated drug metabolism capability of each selected variant with that of WT and the curated reference based on PharmVar to assess the effects of individual variants on drug metabolism.

For the selected 28 genomic variants and WT, recombinant CYP2C19 containing each variant was expressed in Expi293F cells. Since P450 expression was not detected in HEK293 cells, P450-P450 interactions between WT P450 and polymorphic CYP2C19 that reducing the contribution of the polymorphic CYP2C19 could be avoided while using a mammalian cell system (Reed and Backes, 2012; Reed and Backes, 2017). Furthermore, the inhibition of *S*-mephenytoin metabolism by CYP2C9-CYP2C19 interaction could also be avoided (Hazai and Kupfer, 2005; Reed and Backes, 2012; Uhlen et al., 2017). The capability of metabolizing *S*-mephenytoin and omeprazole was measured for the polymorphic CYP2C19 purified from the human microsome and compared to WT.

As determined by immunoblotting with microsomal extracts, polymorphic CYP2C19 containing each of the eight genomic variants (c.556C>T, c.778C>A, c.784G>A, c.1003C>T, c.1034T>A, c.1150G>C, c.1160T>C, and c.1295A>T) were considered that few mature CYP2C19 were present in the endoplasmic reticulum (ER) membrane (Figure 2). This is supported by the fact that CYP2C19 is an integral membrane protein (IMP) (Monier et al., 1988). The nascent chain of the protein is cotranslationally inserted into the ER to become a mature protein in the membrane (Monier et al., 1988). Therefore, in CYP2C19 containing such genomic variants, the possibility that the process of mature protein production is interrupted by an uncertain mechanism has been suggested. When CYP2C19 containing c.218G>A was measured using CO-difference spectroscopy, the absorption peak at approximately 450 nm was not readily detected, despite a sufficient amount of recombinant CYP2C19 in the ER (Figure 2; Table 3). This result indicates that the catalytic cycle has not been initiated (Meunier et al., 2004) and it is considered that the structural change of CYP2C19 containing c.218G>A may interrupt the initiation, leading to the loss of metabolic enzyme activity. Among the 2,504 subjects registered in the 1000 Genomes Project, 17 subjects had c.218G>A variants or the eight genetic variants that interfere with mature protein production. In such cases, lansoprazole or rabeprazole can be prescribed as alternatives except for omeprazole, which is metabolized by *CYP2C19* (Lim et al., 2005; Scott et al., 2014).

The *S*-mephenytoin metabolizing enzyme activity assay confirmed that the polymorphic P450 2C19 protein containing four individual variants with reduced metabolic capability compared to c.431G>A (CYP2C19*9), known as the decreased function allele (Table 3). Metabolites were not detectable in P450 2C19 with c.373C>T or c.394C>T. Therefore, these two variants could be classified as decreased functions. c.389C>T and c.1330G>C may also be considered low-functioning genotypes in *S*-mephenytoin metabolism because their OH-mephenytoin production is only approximately half of that of c.431G>A (CYP2C19*9). Based on the electrophoretic measurement of metabolites of omeprazole, P450 2C19 carrying p c.373C>T, c.394C>T, or c.1330G>C had significantly lower enzyme activities than P450 2C19 carrying c.431G>A (CYP2C19*9) (Table 3). Although P450 2C19 carrying c.431G>A (CYP2C19*9) has a metabolic capability of 63.1% of WT for omeprazole, these three variants could also be classified as genotypes with decreased function since the polymorphic proteins encoded by these variants have a metabolic capability of less than 50% of WT. The structural changes observed in the stereoscopic structure of 4GQS containing each variant were located in the decisive structure for drug discrimination, supporting the functional analysis results (Figures 5A–E, G, H). P450 2C19 containing c.1465C>T displayed 269.0% of WT metabolism to omeprazole (Table 3). CYP2C19*17 increases the metabolizing enzyme activity by nearly twice that of the WT to omeprazole and approximately four times that of *S/R*-mephenytoin (Sim et al., 2006). Therefore, c.1465C>T is suggested as a candidate for an increased function variant, with enzyme activity increased by 2.7 times of WT to omeprazole and 1.8 times to *S*-mephenytoin.

Among polymorphic proteins carrying variants not listed in PharmVar, 75% (18/24) showed significant differences in drug metabolism compared to WT (Figure 3; Table 3). Of these, 11 individual genomic variants were predicted by SIFT and PolyPhen-2 to be damaged and met the CADD exact score of greater than 20 (Figure 3; Table 3). While these three *in silico* prediction algorithms have been primarily applied to detect rare Mendelian disease variants, we found that a certain combination of these *in silico* prediction algorithms can be used to screen candidate variants for substantial changes in drug metabolism, especially for decrease function variants as well as variants with severely lowered expression (Figure 3). Since metabolic enzyme activity analysis of all possible genomic variants in all exon positions of the gene has limitations in terms of time and cost, it is useful to apply prediction methods using *in silico* prediction algorithms. However, despite the effective use of *in silico* prediction algorithms, it is still important to obtain information by metabolizing enzyme activity analysis based on changes in protein structural characteristics and substrate types for CYP proteins to interact with substrates. One reason is that conservation is important in many *in silico* prediction algorithms, whereas the amino acid sequence of the SRSs is not well conserved with other species, which is the most hyperpolymorphic region in CYPs (Gotoh, 1992).

Furthermore, unlike most classical enzymes with strict substrate selectivity, CYPs can each metabolize a number of substrates that differ in size, shape, and stereochemistry (Johnson, 1992; Derayea et al., 2019). This suggests the possibility of different drug-specific metabolic capabilities for one genomic variant, while the existing pharmacogenetic guidelines and *in silico* prediction algorithms generate results without considering the type of substrate. In our study, polymorphic P450 2C19 proteins indicating different tendencies of metabolic capability in the two substrates were detected (5/24 = 20.8%; Table 3 and Figure 3). Of these proteins, the largest metabolic capability difference between the two substrates was observed in the case of polymorphic P450 2C19 containing c.394C>T (71.3%, *p* = .0011), which was found to be lower than that of CYP2C19*9 to *S*-mephenytoin, but similar to WT to omeprazole (*S*-mephenytoin 15.5%, omeprazole 86.8%) (Table 3; Figure 3). The reproduced crystal structures of polymorphic CYP2C19 containing c.389C>T exhibited structural changes in SRS 2, SRS 4, and the active site, which are the key structures for distinguishing drug specifications of CYP2C19 (Evans and Relling, 1999; Tornio and Backman, 2018) (Figures 5C, D). The structural change in the key structures were also observed in the stereoscopic model of polymorphic CYP2C19 containing c.593T>C (*S*-mephenytoin 49.6%, omeprazole 81.2%, *p* = .0017) (Figures 5G, H). P450 families are well known for their ability to metabolize multiple substrates, and each substrate has its own sites for interaction with CYP2C19, even chemically homologous drugs have different binding specificity and CYP-drug interaction due to their tiny structural differences (Ibeanu et al., 1996; Payne et al., 1999; Derayea et al., 2019). Therefore, the possibility of different drug-specific metabolic capabilities for single genomic variant is supported by our metabolic capability assay using the two substrates and the crystal structure analysis of polymorphic CYP2C19.

A limitation of this study is that the metabolizing enzyme activity assay for this approach is a one-at-a-time functional assay requiring a relatively long time and is labor-intense. In particular, it is impossible to determine the drug metabolic capability to vary by substrate because *in silico* protein damage prediction algorithms generate results that do not consider the type of substrate. High-throughput expression screening assays might also have advantages over conventional functional analysis in that they reduce the intensity of time and labor; however, further advancement is still needed to investigate drug metabolism (Vanoye et al., 2018; Glazer et al., 2020; Zhang et al., 2020). It is also difficult to comprehensively predict changes in metabolic capabilities over different multiple variant combinations and impossible to analyze metabolic capabilities considering changes in amino acid at specific genomic position by each isoform. However, since *CYP2C19* has only one isoform, our results for each genomic variant are suitable for providing functional information supporting the existing guidelines.

In summary, we characterized the metabolic enzyme activity of *CYP2C19* genomic variants with *in vitro* and *in silico* assessments using *S*-mephenytoin and omeprazole. All spontaneously present variants were synthesized using HEK293 cells, which allowed us to use the human cell system without reducing the contribution of the polymorphic CYP2C19. The results verified that there are polymorphic proteins containing genomic variants with significantly changed metabolizing capabilities compared to that of the WT, although they have yet to be considered in existing pharmacogenetic guidelines. It was also confirmed that there are polymorphic proteins with different disposition of metabolizing enzyme activity depending on substrates. These genomic variants causing changes in metabolic capabilities analyzed in this study will be reported to the PharmVar Database to be evaluated in the updating CPIC guidelines and proposed as a priority consideration for clinical testing. Therefore, we propose a methodology that combines analysis methods using functional assays on CYP protein correlated with binding to multiple substrates and prediction methods using *in silico* prediction algorithms to evaluate the changes in the metabolism of all possible genomic variants in CYPs. Further research on both functional analysis methods and *in silico* prediction algorithms is needed to improve measurement accuracy, and the classification criteria re-established through the process would reinforce existing guidelines and provide information for prescribing appropriate medicines for individual patients.

## Data Availability

The datasets presented in this study can be found in online repositories. The names of the repository/repositories and accession number(s) can be found in the article/Supplementary Material.
