# Supplementary figures and images for: Comprehensive in vitro and in silico assessments of metabolic capabilities of 24 genomic variants of CYP2C19 using two different substrates
Source: Front Pharmacol. 2023 Jan 12;14:1055991. doi: 10.3389/fphar.2023.1055991 (PMC9877350; doi:10.3389/fphar.2023.1055991)

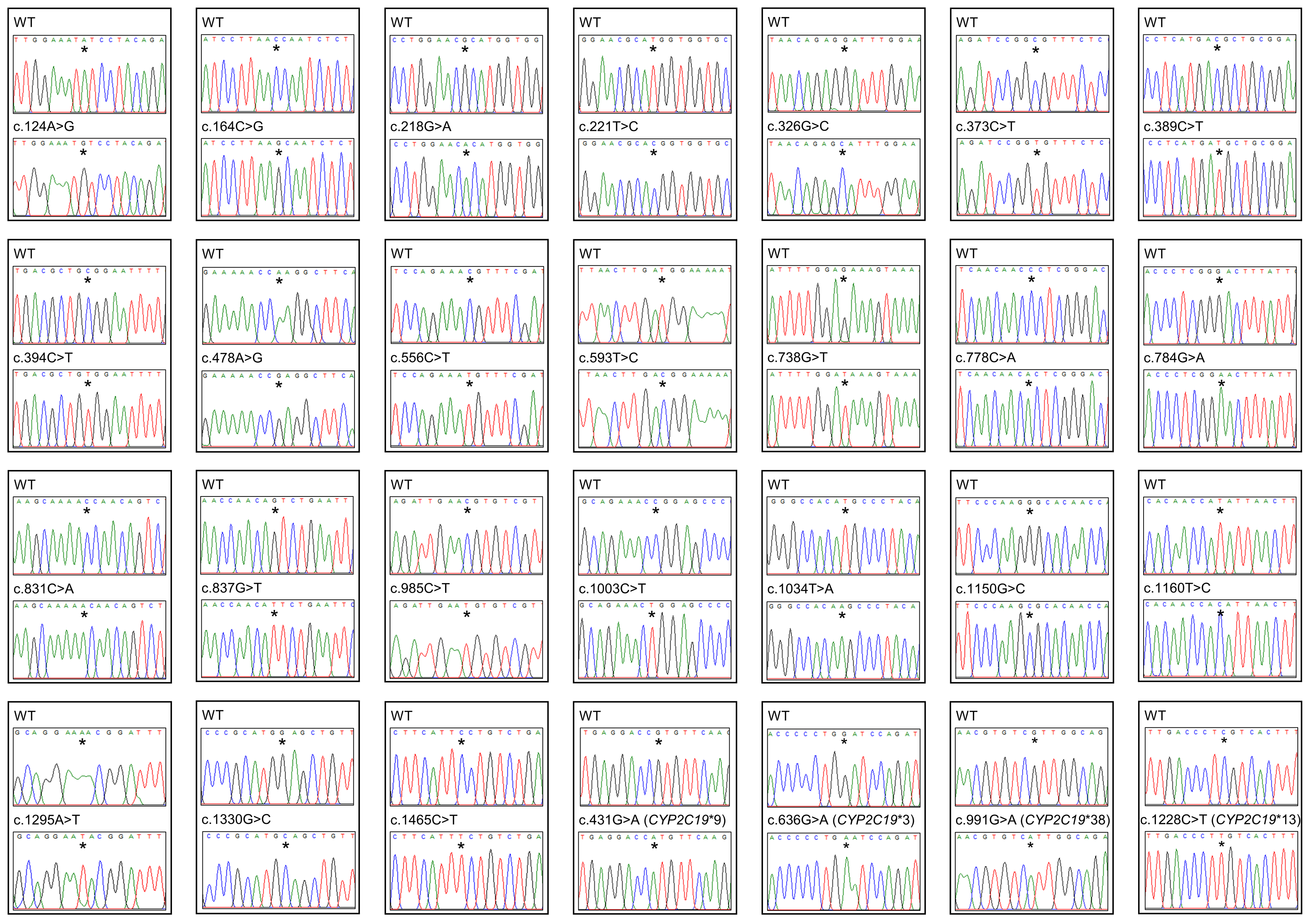

Supplement: Supplementary file 1 [file Image1.TIF]
